# Schizophrenia liability shares common molecular genetic risk factors with sleep duration and nightmares in childhood

**DOI:** 10.12688/wellcomeopenres.15060.2

**Published:** 2019-08-20

**Authors:** Zoe E. Reed, Hannah J. Jones, Gibran Hemani, Stanley Zammit, Oliver S. P. Davis

**Affiliations:** 1 Medical Research Council Integrative Epidemiology Unit, University of Bristol, Bristol, UK; 2 Department of Population Health Sciences, Bristol Medical School, University of Bristol, Bristol, UK; 3 Centre for Academic Mental Health, Department of Population Health Sciences, Bristol Medical School, University of Bristol, Bristol, UK; 4 MRC Centre for Neuropsychiatric Genetics and Genomics, Institute of Psychological Medicine and Clinical Neurosciences, Cardiff University, Cardiff, UK

**Keywords:** polygenic risk, genetic correlation, schizophrenia, sleep, childhood, ALSPAC

## Abstract

**Background: **Sleep abnormalities are common in schizophrenia, often appearing before psychosis onset; however, the mechanisms behind this are uncertain. We investigated whether genetic risk for schizophrenia is associated with sleep phenotypes.

**Methods: **We used data from 6,058 children and 2,302 mothers from the Avon Longitudinal Study of Parents and Children (ALSPAC). We examined associations between a polygenic risk score for schizophrenia and sleep duration in both children and mothers, and nightmares in children, along with genetic covariances between these traits.

**Results: **Polygenic risk for schizophrenia was associated with increased risk of nightmares (OR=1.07, 95% CI: 1.01, 1.14, p=0.02) in children, and also with less sleep (β=-44.52, 95% CI: −88.98, −0.07; p=0.05). We observed a similar relationship with sleep duration in mothers, although evidence was much weaker (p=0.38). Finally, we found evidence of genetic covariance between schizophrenia risk and reduced sleep duration in children and mothers, and between schizophrenia risk and nightmares in children.

**Conclusions:** These molecular genetic results support recent findings from twin analysis that show genetic overlap between sleep disturbances and psychotic-like experiences. They also show, to our knowledge for the first time, a genetic correlation between schizophrenia liability and risk of nightmares in childhood.

## Introduction

Sleep disturbance is very common in psychotic disorders such as schizophrenia. For example, a meta-analysis of observational studies showed that sleep efficiency and total sleep time are diminished in schizophrenia, whilst sleep latency is increased, independent of medications ^1^. These findings are supported by polysomnography studies ^2^. Similarly, sleep dysfunction is more common in individuals at ultra-high-risk for psychosis ^3^, and is associated with higher risk of incident psychotic experiences ^4^, paranoid thinking ^5^ and hallucinatory experiences ^6^ in population-based cohort studies. Sleep disturbance includes a range of phenotypes, such as decreased sleep duration, which is associated with schizophrenia ^7^ and nightmares which is associated with psychotic experiences and patients with early psychosis ^4, 8, 9^.

A recent report from the Twins Early Development Study (TEDS) demonstrated that psychotic-like experiences and sleep disturbances in adolescence, around the age of 16 years, share genetic influences ^10^. Using data from 4,800 pairs of twins, they found a mean genetic correlation of 0.54 between self-reported paranoia, hallucinations and cognitive disorganisation, and self-reported sleep quality and insomnia. Bivariate heritability analyses showed that shared genetic influences accounted for on average 65% of the phenotypic correlation between phenotypes. This genetic relationship could be explained in several ways. For example, genetic variants could independently influence sleep and psychotic-like experiences, both sleep and psychotic-like experiences could be affected by a shared genetically influenced process, or there could be a causal relationship between sleep and psychotic-like experiences. We aimed to build on these analyses by using a polygenic risk score derived from the largest genome-wide association study (GWAS) of schizophrenia to date ^11^ to investigate the genetic relationship between schizophrenia liability, sleep duration and childhood nightmares through two complimentary approaches.

First, we examined whether genetic risk for schizophrenia is associated with sleep dysfunction during childhood and adulthood in the population-based Avon Longitudinal Study of Parents and Children (ALSPAC). We did this by combining information from the Psychiatric Genomics Consortium (PGC) GWAS into a polygenic risk score that indexes cumulative genetic risk for schizophrenia. The PGC report a variance explained in schizophrenia by the polygenic risk score at a p-value threshold of 0.05 of 7% ^11^. This approach has previously shown that genetic risk for schizophrenia is associated with phenotypes such as cognitive deficits ^12, 13^, anxiety and negative symptoms ^14^ during childhood and adolescence.

Second, we assessed the genetic covariance between schizophrenia and sleep measures in childhood and adulthood using polygenic risk score data to estimate the average genome-wide relationship between these phenotypes.

## Methods

### Cohort description

ALSPAC is a prospective birth cohort study which initially recruited 14,541 pregnant women living in the Bristol area in the UK, with an expected delivery date from 1 ^st^ April 1991 to the 31 ^st^ December 1992 ^15, 16^. A total of 14, 676 foetuses were included in the initial ALSPAC sample, with 14,062 live births and, of these, 13,988 were alive after 1 year. At 7 years of age further recruitment occurred, resulting in a total sample size for analyses of 15,458, with 14,755 live births and 14,701 alive at 1 year. Data has been collected on mothers and their offspring via questionnaires, clinic visits and other forms of information. The ALSPAC sample is generally representative of the UK population of the same age, although, like many other cohorts, there is over-representation of more affluent groups (6.22% with low household income, as indicated by free school meals, in ALSPAC compared to 12.49% in the National Pupil Database) and under-representation of non-White minority ethnic groups (96.09% White ethnicity in ALSPAC compared to 86.50% in the National Pupil Database) ^15^. However, to avoid false positive results from population stratification, our analyses included only those with European ancestry. We also excluded those without both the genetic and the phenotypic data required, leaving between 5,121 and 6,058 children and 4,906 mothers in each analysis. Table 1 summarises the number of individuals with data available for each of the outcome measures. Including all the time points examined for sleep duration, there were a total of 28,138 data points.

**Table 1.** Number of genotyped participants with each outcome measure.

Time point	Outcome measure	N ^1^	Percentage female	Mean age (SD)	Mean outcome measure or percentage
*Children*					
4 years 9 months	Sleep duration	6058	48.6%	4.78 (0.10)	11.39 (0.71)
5 years 9 months	Sleep duration	5641	48.9%	5.80 (0.11)	11.29 (0.69)
6 years 9 months	Sleep duration	5534	48.9%	6.78 (0.11)	11.14 (0.66)
9 years 7 months	Sleep duration	5735	49.4%	9.65 (0.12)	10.44 (0.66)
11 years 8 months	Sleep duration	5170	50.2%	11.72 (0.13)	9.80 (0.71)
12 years	Nightmares	5121	51.1%	12.81 (0.23)	Absent = 74.97%, Suspected = 8.87%, Present = 16.17%
*Mothers*					
Average age of 40 years 6 months	Sleep duration	4906	100%	40.48 (4.53)	7.37 (0.99)

^1^The number of participants with both phenotype and genotype data available

Further details of ALSPAC can be found through the searchable data dictionary. Ethical approval for the study was obtained from the ALSPAC Ethics and Law Committee and the local Research Ethics Committees. Written informed consent was obtained from participants or parents of participants, for children. Children were invited to give assent where appropriate. Study members have the right to withdraw their consent for elements of the study or from the study entirely at any time. Full details of the ALSPAC consent procedures are available on the study website ( http://www.bristol.ac.uk/alspac/researchers/research-ethics/).

### Phenotypic measures

***Sleep duration*.** Information about sleep duration in children was obtained from parent-completed questionnaire data collected at the ages of 4, 5, 6, 9 and 11 years. Sleep duration was calculated as the difference between reported time that the child went to sleep and time they awoke on weekdays.

Sleep duration in the mothers was assessed through questionnaire data collected when they were on average 40.48 years old (SD=4.53). Participants were asked how many hours and minutes they slept for on work days on average in the last year.

***Nightmares*.** The presence of nightmares was assessed through semi-structured interviews with participants at around the age of 12 and a half years ^4, 8^. Briefly, children were asked questions about nightmares, night terrors and sleepwalking since their 12 ^th^ birthday, such as *“Since your 12 ^th^ birthday have you had any dreams that woke you up? Were they frightening?”*. Positive responses were followed by further questions to distinguish between nightmares and night terrors. These were then rated as present, suspected or absent. In this study we use data on nightmares only as this was associated with psychotic experiences in a previous study ^8^, and we wanted to examine whether this extended to genetic risk for schizophrenia.

***Covariates*.** We used the child’s sex and age as covariates in the analyses of children’s sleep outcomes. We included mother’s age as a covariate in the analyses of mother’s sleep duration. We did not control for covariates beyond age and sex within the analyses as these would fall temporally between the independent variables (polygenic scores) and outcome (sleep) and could therefore act as mediators. Adjusting for these potential mediators could lead to misleading results.

### Genetic data

Genetic data for children and mothers was collected in the form of blood samples during clinic visits. Genotyping for children was conducted using the Illumina HumanHap 550quad chip and data was generated by Sample Logistics and Genotyping Facilities at the Wellcome Trust Sanger Institute and LabCorp (Laboratory Corporation of America) using support from 23andMe. ALSPAC mothers were genotyped using the Illumina human660W-quad array at Centre National de Génotypage (CNG) and genotypes were called with Illumina GenomeStudio.

Quality control measures were conducted, and SNPs were excluded based on missingness, Hardy-Weinberg equilibrium P value and minor allele frequency. Samples were excluded based on gender mismatches, indeterminate X chromosome, minimal or excessive heterozygosity, disproportionate missingness, insufficient sample replication and evidence of population stratification. A total of 9,115 children and 500,527 SNPs passed the filters and data was imputed with a phased version of the 1000genomes reference panel from the Impute2 reference data repository. 9,048 mothers and 526,688 SNPs passed the filters and data was imputed using Impute V2.2.2 against the reference panel (all polymorphic SNPs excluding singletons), using all 2186 reference haplotypes (including non-Europeans). After these procedures, removing participants with cryptic relatedness >5% and those who had withdrawn consent, there were 8,252 children and 8,252 mothers with genotype data available.

### Polygenic risk score construction

We constructed polygenic risk scores using Plink version 1.9 ^17^ for each individual using summary statistics from the PGC schizophrenia GWAS ^11^. SNPs with an imputation quality score greater than 0.9 and which were available in ALSPAC were clumped for linkage disequilibrium (LD), using an R ^2^ of 0.25 and a window of 500kb. We generated weighted polygenic risk scores by summing the number of risk alleles present for each SNP (0, 1 or 2) weighted by the logarithm of its discovery sample odds ratio (OR). For estimating covariance (see below) we constructed twelve polygenic risk scores based on different thresholds of the schizophrenia GWAS p-values (1×10 ^−7^ to 0.5). The score constructed with a p-value threshold (pT) of 0.05 captured the most variance in genetic liability for schizophrenia in the PGC sub-datasets ^11^, so we used this as our primary exposure, with the remainder reported in Extended data Table 1 and Extended data Figure 1 ^18^. Polygenic risk scores were z-standardised, so effect sizes are given per standard deviation (SD) increase in polygenic risk score.

### Statistical analysis

***Association between polygenic risk score and sleep phenotypes*.** We conducted statistical analyses in R version 3.2.2 ^19^ and Stata version 14.2 ^20^. First, we investigated the association between the polygenic risk score for schizophrenia liability and sleep duration in childhood, by combining all time points into one stacked dataset. We used a linear mixed-effects model, with age nested within family ID as a random effect and covariates age, age ^2^ and sex. We tested for an association between the polygenic risk score for schizophrenia liability and nightmares using an ordinal model with sex as a covariate. We tested the association between the polygenic risk score for schizophrenia liability and sleep duration in mothers using a linear model with age as a covariate. To estimate the variance explained by the schizophrenia polygenic risk score at pT 0.05 for each outcome we obtained r-squared values from models without covariates; r-squared was obtained from the linear model, a pseudo r-squared was obtained from the ordinal model and a marginal r-squared (the proportion of variance explained by the fixed factors) was obtained for the linear mixed-effects model.

***Estimating genetic covariance between schizophrenia liability and sleep phenotypes in childhood and adulthood*.** Because individual level data were available for the sleep phenotypes and summary data were available for the schizophrenia phenotype, we used the Additive Variance Explained and Number of Genetic Effects Method of Estimation (AVENGEME) ^21^ to estimate the genetic covariance between schizophrenia liability and sleep duration and schizophrenia liability and nightmares in children, and between schizophrenia liability and sleep duration in mothers. To estimate a genetic model AVENGEME used the T-scores from results of the association between a series of polygenic risk scores at different discovery sample thresholds and the outcomes, sample sizes from the training and test samples, the number of variants used to create the scores, the p-value thresholds used to create the scores and the population prevalence of schizophrenia (we used 0.04) and case/control sampling fractions for the PGC data (0.43). Using this information, the approach is able to estimate the variance explained by genetic effects in the training sample, the variance explained by the polygenic risk score in the outcomes in ALSPAC, the genetic covariance between the training and target samples and the proportion of null SNPs with no effect on the trait in the training sample.

## Results

### Association between polygenic risk of schizophrenia and sleep phenotypes

***Sleep duration in children*.** We found that a 1SD increase in the schizophrenia polygenic risk score was associated with a decrease in sleep duration of 44.52 seconds (95% CI: −88.98, −0.07; p=0.05) ( Table 2). This was consistent across other polygenic risk score thresholds (pT 0.01–0.5) ( Extended data, Table 1 and Extended data, Figure 1 ^18^). Examining sleep duration at different ages indicates that the effect size increases after age 5 ( Extended data, Table 2 ^18^), although there was little evidence of an interaction between polygenic risk and age when an interaction term was added to the mixed-effects model (p=0.23).

**Table 2.** Associations between schizophrenia polygenic risk (pT 0.05) and sleep outcomes.

Sleep phenotype (units)	Beta or OR	95% CI	R-squared ^1^	*P*-value
Sleep duration in children (seconds) ^2^	-44.52	-88.98, -0.07	0.0002	0.05
Nightmares in children (ordinal, OR)	1.08	1.01, 1.14	0.001	0.02
Sleep duration in mothers (seconds)	-49.97	-150.48, 50.55	0.0003	0.33

^1^The r-squared values were a marginal r-squared (the proportion of variance explained by the fixed effects) for sleep duration in children, Nagelkerke’s pseudo r-squared for nightmares and the r-squared for sleep duration in mothers. ^2^For this analysis we combined all time points into one stacked dataset in a linear mixed-effects model, resulting in 28,138 data points in the model.

***Nightmares in children*.** We found evidence of increased risk of nightmares in those with greater polygenic risk (OR=1.07; 95% CI: 1.01, 1.14; p=0.02) ( Table 2). We found a similar pattern for other polygenic risk score thresholds (0.05–0.5) ( Extended data, Table 4 and Extended data Figure 2 ^18^). We found no evidence that the proportional odds assumption was violated (p=0.89).

Adjusting for the mothers’ polygenic risk score in the analyses for sleep duration and nightmares in children had minimal effect ( Extended data Tables 3 and 5 ^18^), indicating that the association is not confounded by the mother’s genetic liability to schizophrenia.

***Sleep duration in mothers.*** The estimated effect size of the association between schizophrenia polygenic risk score and sleep duration in mothers was similar to that seen in children, although confidence intervals are wide, so evidence in support of this association was weak (β=-49.97 seconds; 95% CI: −150.48, 50.55; p=0.33) ( Table 2). The smaller sample size in the mothers’ cohort has most likely led to lower power and therefore less precise estimates. An increased sample size would allow for estimation of this effect with greater precision. The results in mothers for other polygenic risk score thresholds can be seen in Extended data Table 6 and Extended data Figure 3 ^18^, where scores calculated at more stringent p-value thresholds showed small positive associations (1×10 ^-7^ to 0.01) compared with small negative associations for pT 0.5 to 0.01.

## Genetic overlap between sleep phenotypes and schizophrenia risk

In the ALSPAC children, our AVENGEME analyses indicate a positive covariance between nightmares and genetic liability for schizophrenia (covariance= 0.07, 95% CI: 0.05, 0.10). We found a negative covariance between sleep duration and genetic liability for schizophrenia in children (covariance= −0.008, 95% CI: −0.20, −0.003) and between sleep duration and genetic liability for schizophrenia in mothers (covariance= −0.004, 95% CI: −0.03, −0.0001) (see Extended data Table 7 ^18^ for full results).

## Discussion

We investigated whether genetic risk for schizophrenia is associated with sleep outcomes during childhood and adulthood. In this study we used summary statistics from a schizophrenia GWAS of an adult, clinical sample to construct polygenic risk scores in children and adults in the aim of understanding how schizophrenia genetic liability manifests in childhood. Our results demonstrate, for the first time with molecular genetic data, that greater polygenic risk for schizophrenia is associated with shorter sleep duration in both childhood and adulthood, and we report a new association between greater polygenic risk of schizophrenia and increased risk of nightmares in early life.

These results build on previous twin study findings of shared genetic effects between schizophrenia and disrupted sleep ^10^ by showing that the association extends to collections of known and measured DNA variants identified through recent well-powered GWAS studies. In addition, finding a small to moderate association between greater polygenic risk of schizophrenia and increased risk of nightmares supports previous phenotypic observations between nightmares and psychotic experiences suggesting the presence of nightmares may be an early risk indicator for psychosis ^4, 8, 9^ and suggesting that this relationship could be partly genetically mediated.

The effect size of schizophrenia genetic risk on sleep duration in childhood in this study is small, equating to only a few minutes of sleep difference between individuals scoring three standard deviations above, compared to individuals scoring three standard deviations below the mean for polygenic risk. This is likely to reflect, in part, measurement error in the estimation of the effect sizes of the individual DNA variants that make up the polygenic score, and the fact that the pool of variants for the polygenic score is itself an incomplete set of human genomic variation, where many of the variants are imperfect proxies for true causal variants.

We found that the schizophrenia polygenic risk score explains only 0.02% of the variation in sleep duration. However, the best available polygenic risk score for schizophrenia only accounts for 7% of the variance in schizophrenia itself ^11^, so we would be unlikely to find that the PRS accounted for more variance than this in a related phenotype such as sleep. In cross-disorder analyses, the schizophrenia polygenic risk score explains variation ranging from 0.08% for autism spectrum disorder to 2.3% for bipolar disorder ^22^, with similarly low variance explained for cognition (<1%) ^13^. Therefore, whilst we find a small amount of variation explained in sleep duration, this is on a similar scale to other cross-disorder analyses.

Small effect sizes may also reflect measurement error and quality of the parent-reported outcome measures. Self-reported sleep duration is subjective and therefore there will be some measurement error introduced when reporting average sleep duration, so the effect sizes we report here may underestimate the true correlations. This measurement error would also impact the parent-reported measures of sleep in their children, though we opted to use child’s sleep duration on weekdays only and not on weekends to capture routine sleep patterns.

Although we combined measures of sleep duration from several different ages, which is likely to have reduced error that was not shared across measurement occasions, sleep duration in the mothers is reported at a single time point and is unlikely to be consistent throughout the life course. The question for mother’s sleep duration also asks specifically about sleep on work days which may result in a bias if individuals who do not work did not answer this question. However, using data from another question asking, “Have you had any jobs or regular voluntary work in the past year?”, we found that of those who responded “yes” to this question, 98.64% responded to the question about sleep on work days. Of those who responded “no” to this question, 94.65% responded to the question about sleep on work days. These are quite similar response rates and although the latter is slightly smaller, we believe that this impact is negligible. More reliable measures of sleep duration are not currently available in the ALSPAC cohort, however, future studies comparing self-report measures to objective measures (such as accelerometer-derived sleep estimates ^23^) may help to estimate the potential biases in our results. Similarly, although we have reported results for sleep duration, we have no direct measure of sleep quality, so the marginally lower effect size for duration of sleep may index greater disruption in sleep architecture that could have important effects on psychopathology.

Although the effect sizes we report imply limited clinical relevance, the aforementioned limitations on our measurement of both sleep and genetic risk for schizophrenia provide a practical upper bound for the effect size we could have found. As schizophrenia is highly heritable, better-powered GWAS will inevitably identify further variants and more accurately estimate genetic effect sizes, resulting in polygenic scores that account for a greater proportion of the variance. In addition, in our study we were unable to assess individual domains of schizophrenia, for example hallucinations, delusions or negative symptoms, as there are currently no available well powered GWAS for these outcomes. Future studies that reduce measurement error through objective measurements of sleep and more comprehensive polygenic risk scores that, for example, index specific symptom domains as well as disorder, will likely estimate more accurate and larger effect sizes than those we report here. As such, it is probably unwise to rule out clinical relevance on the basis of current effect size estimates.

Whilst these findings provide evidence of some underlying shared genetic mechanism between sleep phenotypes and schizophrenia, they do not allow us to determine the reason behind these associations. For example, the findings could be due to horizontal pleiotropy, whereby genetic variants associated with schizophrenia are also independently associated with sleep phenotypes. The findings could also be due to causality, where either liability to schizophrenia causes disruption to sleep, or sleep disruption causes schizophrenia and so variants associated with sleep phenotypes are captured by the schizophrenia GWAS, which seems to be supported by experimental evidence ^24^. It is also possible that the polygenic risk score for schizophrenia may capture a shared risk factor for both schizophrenia and sleep disruption, e.g. urbanicity, and so the results represent the effect of this unknown shared risk factor on both phenotypes. Finally, associations may also be induced by population stratification where spurious results arise due to differences in genetic backgrounds between the discovery and target samples (although this is less likely due to restriction to those with a European ancestry). However, we also acknowledge that given that the effects detected in the current study are small, investigating underlying mechanisms between sleep factors and schizophrenia would be difficult. Objective sleep measures and more complete knowledge of the genetic variants related to sleep and schizophrenia would likely increase power to explore mechanisms.

Recent studies indicate that there may be a causal relationship. For example, a trial of cognitive-behavioural therapy for insomnia in people with psychosis reported a decrease in persecutory delusions, hallucinations, anxiety and depression ^25^, suggesting a causal effect of sleep dysfunction on psychopathology post-onset of psychosis. However, another study reported more mixed results with psychosis symptoms ^26^, and effects of sleep disturbance on incidence of psychosis might differ from those on illness severity. Randomised control trials that examine the impact of sleep interventions on psychosis incidence are not feasible given the incidence rate of psychotic disorders. Our results seem to mostly suggest the direction described in the first study, where disturbed sleep may result in increased risk for schizophrenia as our study finds associations with disturbed sleep in childhood and adolescence prior to any disorder and the association is with schizophrenia risk. However, it is possible that the reverse direction could be true if schizophrenia risk results in sub-clinical symptoms that cause disturbed sleep. There could also be a bi-directional causal association present or no causal association at all. Future well-powered analyses using techniques such as Mendelian randomisation will be useful for examining whether relationships are causal and the direction of causality. It would also be interesting to see whether the same relationship between polygenic risk for schizophrenia and sleep duration is observed in adult males, as were only able to examine this in adult females here.

## Conclusion

Our results use molecular data to support and extend the findings from twin studies of an overlap of genetic influences on sleep phenotypes and schizophrenia. We have presented novel findings with our use of polygenic risk scores that specifically relate to liability for schizophrenia risk, as opposed to psychotic experiences. In addition, we found a novel relationship of shared genetic influences between schizophrenia and nightmares in children.

## Data availability

### Underlying data

The ALSPAC data management plan ( http://www.bristol.ac.uk/alspac/researchers/data-access/documents/alspac-data-management-plan.pdf) describes in detail the policy regarding data sharing, which is through a system of managed open access. The steps below highlight how to apply for access to the data included in this paper and all other ALSPAC data. The datasets used in this analysis are linked to ALSPAC project number B2172; please quote this project number during your application.

1. Please read the ALSPAC access policy (PDF, 627 kB) which describes the process of accessing the data and samples in detail, and outlines the costs associated with doing so.
2. You may also find it useful to browse the fully searchable ALSPAC research proposals database, which lists all research projects that have been approved since April 2011.
3. Please submit your research proposal for consideration by the ALSPAC Executive Committee. You will receive a response within 10 working days to advise you whether your proposal has been approved.

If you have any questions about accessing data, please email alspac-data@bristol.ac.uk.

### Extended data

Extended data Tables 1–7 and Figures 1–3 are available via the Open Science Framework: Schizophrenia liability shares common molecular genetic risk factors with sleep duration and nightmares in childhood. DOI: http://doi.org/10.17605/OSF.IO/HPZ5Y ^18^.

Supplementary Table 1. Associations between standardised schizophrenia polygenic risk score and sleep duration in children in ALSPAC.

Supplementary Table 2. Results from linear regressions for sleep duration and polygenic risk score for schizophrenia at each of five time points in childhood in ALSPAC.

Supplementary Table 3. Associations between standardised schizophrenia polygenic risk score and sleep duration in children in ALSPAC, whilst adjusting for mother’s polygenic risk at the same threshold.

Supplementary Table 4. Associations between standardised schizophrenia polygenic risk score and nightmares in children in ALSPAC.

Supplementary Table 5. Associations between standardised schizophrenia polygenic risk score and nightmares in children in ALSPAC, whilst adjusting for mother’s schizophrenia polygenic risk score at the same threshold.

Supplementary Table 6. Associations between schizophrenia polygenic risk score and sleep duration in mothers in ALSPAC.

Supplementary Table 7. Results from estimating a polygenic model using AVENGEME, with T-scores from the observational associations and the same 12 thresholds used for the polygenic risk score construction.

Supplementary Figure 1. Associations between polygenic risk score for schizophrenia at 12 different p-value thresholds and sleep duration in children in ALSPAC.

Supplementary Figure 2. Associations between polygenic risk score for schizophrenia at 12 different p-value thresholds and nightmares in children in ALSPAC.

Supplementary Figure 3. Associations between polygenic risk score for schizophrenia at 12 different p-value thresholds and sleep duration in mothers in ALSPAC.

Data are available under the terms of the Creative Commons Zero "No rights reserved" data waiver (CC0 1.0 Public domain dedication).
