# Supplementary material for: Schizophrenia liability shares common molecular genetic risk factors with sleep duration and nightmares in childhood
Source: Wellcome Open Res. 2019 Aug 20;4:15. Originally published 2019 Jan 25. [Version 2] doi: 10.12688/wellcomeopenres.15060.2 (PMC6753602; doi:10.12688/wellcomeopenres.15060.2)
Supplement: Peer review history

# Peer review: Reviewer response for version 2

**DOI:** 10.21956/wellcomeopenres.16848.r36264

**Published:** 2019-09-18

**Authors:** Bryony Sheaves

**Affiliations:** 1 Department of Psychiatry, University of Oxford, Oxford, UK

Thank you for addressing the points in the previous review, I enjoyed reading the revised manuscript. There are two minor points for consideration:

1. “specific symptom domains as well as disorder, will likely estimate more accurate and larger effect sizes than those we report here.”
  I'm afraid I disagree that indexing specific symptoms will lead to larger effect sizes. In fact initial evidence from the Reeve *et al*. study (2017, already referenced in a different context) indicated that restricting sleep duration significantly increased paranoid thoughts and hallucinatory experiences, but not grandiosity. This suggests that the relationships might differ across specific symptoms, but are not necessarily bigger. 
2. “a trial of cognitive-behavioural therapy for insomnia in people with psychosis reported a decrease in persecutory delusions, hallucinations, anxiety and depression ^25^”.
  The reference here is to a trial protocol, not reporting results. Are you referring to the definitive OASIS trial (Freeman *et al.*, 2017 ^1^) or the initial pilot work by Myers *et al.* (2011 ^2^)? You may have also seen our very recently published pilot RCT treating nightmares in psychosis (Sheaves *et al.*, 2019 ^3^). We observed moderate effect size improvements in paranoia after treating nightmares (albeit with wide confidence intervals). It was not powered to detect significant changes in psychotic experiences so may not warrant referencing, but I’ve mentioned in case it is of interest.

I confirm that I have read this submission and believe that I have an appropriate level of expertise to confirm that it is of an acceptable scientific standard.

## References

[1] : The effects of improving sleep on mental health (OASIS): a randomised controlled trial with mediation analysis. *The Lancet Psychiatry*.2017;4(10) : 10.1016/S2215-0366(17)30328-0 749-758 10.1016/S2215-0366(17)30328-0 PMC561477228888927

[2] : Cognitive behavioural treatment of insomnia in individuals with persistent persecutory delusions: a pilot trial. *J Behav Ther Exp Psychiatry*.2011;42(3) : 10.1016/j.jbtep.2011.02.004 330-6 10.1016/j.jbtep.2011.02.004 21367359PMC3566479

[3] : Cognitive Behavioural Therapy for Nightmares for Patients with Persecutory Delusions (Nites): An Assessor-Blind, Pilot Randomized Controlled Trial. *Can J Psychiatry*.2019; 10.1177/0706743719847422 706743719847422 10.1177/0706743719847422 31129983PMC6783669

# Peer review: Reviewer response for version 2

**DOI:** 10.21956/wellcomeopenres.16848.r36265

**Published:** 2019-09-06

**Authors:** Victoria Garfield

**Affiliations:** 1 MRC Unit of Lifelong Health and Ageing at UCL, Institute of Cardiovascular Science, London, UK

I have no further comments to make, as I am satisfied with authors' responses. They have worked hard to address my previous concerns and have done a good job.

I confirm that I have read this submission and believe that I have an appropriate level of expertise to confirm that it is of an acceptable scientific standard.

# Peer review: Reviewer response for version 1

**DOI:** 10.21956/wellcomeopenres.16430.r35123

**Published:** 2019-04-30

**Authors:** Bryony Sheaves

**Affiliations:** 1 Department of Psychiatry, University of Oxford, Oxford, UK

Thank you for the opportunity to review this manuscript which seeks to address whether molecular genetic risk for schizophrenia is associated with both sleep duration and nightmares in the ALSPAC sample. Sleep disruption is common in samples with psychosis, so examining a potential genetic association is a sensible line of inquiry. The manuscript is well written. The below points are for consideration, and primarily relate to contextualising the research among the wider sleep and psychosis literature.

 Introduction:

- It is currently unclear why i) sleep duration and ii) nightmares specifically have been chosen as markers of sleep dysfunction. Introducing the reader to literature on what is already known about sleep dysfunction in clinical samples with psychosis is important to help readers understand the clinical presentation under investigation and choice of measures.

Methods:

- What is the rationale for the choice of covariates?
- The sleep duration data uses only weekday sleep which may be confounded by chronotype. Those who are ‘evening types’ may be more prone to longer sleep duration at the weekend to ‘catch up’ on lost hours of sleep during the week (social jet lag), but this would be missed by the current measurement method for sleep duration. Was there a rationale for using only weekday sleep? If the choice was driven by the data available then this should be acknowledged as a limitation.

Results:

- I think the phrasing for the result of ‘sleep duration in mothers’ may be unintentionally misleading. Whilst the effect size may be similar, the confidence intervals are wide, suggesting the effect size is imprecisely estimated (as the authors acknowledge). At the moment the manuscript reads as if increasing the sample size would improve the evidence in support of the association. It wouldn’t necessarily, but it would allow estimation of the effect with greater precision.

Discussion:

- There is little discussion about the result linking nightmares and schizophrenia. It would be helpful to discuss the size of the effect, and contextualise the result within relevant existing literature linking nightmares with psychosis.
- A study which may help address whether the results are due to pleiotropy is an experimental study by Reeve *et al.* (2018 ^1^) which manipulated sleep duration and assessed the impact on psychotic like experience. 
- Given evidence that there are differing levels of heritability for individual psychotic experiences, is the research limited by the fact that it has assessed risk for schizophrenia as a whole?

I confirm that I have read this submission and believe that I have an appropriate level of expertise to confirm that it is of an acceptable scientific standard, however I have significant reservations, as outlined above.

## References

[1] : Disrupting Sleep: The Effects of Sleep Loss on Psychotic Experiences Tested in an Experimental Study With Mediation Analysis. *Schizophrenia Bulletin*.2017; 10.1093/schbul/sbx103 10.1093/schbul/sbx103 28981834PMC5890488

# Response

**Published:** 2019-08-13

**Authors:** Zoe Reed

**Affiliations:** University of Bristol, UK

Introduction:

- It is currently unclear why i) sleep duration and ii) nightmares specifically have been chosen as markers of sleep dysfunction. Introducing the reader to literature on what is already known about sleep dysfunction in clinical samples with psychosis is important to help readers understand the clinical presentation under investigation and choice of measures.

**Response: **The sleep duration phenotype was selected due to repeated measures of this over time, which gave us greater power to detect an effect. It was therefore the most accurate measure we had available in ALSPAC for sleep quality. We selected the nightmare phenotype due to recent research within ALSPAC suggesting an association between nightmares and psychotic experiences (Fisher *et al.*, 2014). Sleep duration and nightmares have both been found to be associated with schizophrenia in previous studies. We have added the following text to the introduction (paragraph 1):

“Sleep disturbance includes a range of phenotypes, such as decreased sleep duration, which is associated with schizophrenia ^26^ and nightmares which is associated with psychotic experiences and patients with early psychosis ^4, 13, 25^.”

Methods:

- What is the rationale for the choice of covariates?

**Response:** We have included just age and sex as covariates as other covariates would fall, temporally, between the polygenic score and the sleep outcomes and may act as mediators so adjusting for these could lead to misleading results. We have added the following text to the covariates section in the methods to clarify this:

“We did not control for covariates beyond age and sex within the analyses as these would fall temporally between the independent variables (polygenic scores) and outcome (sleep) and could therefore act as mediators. Adjusting for these potential mediators could lead to misleading results.”

 

- The sleep duration data uses only weekday sleep which may be confounded by chronotype. Those who are ‘evening types’ may be more prone to longer sleep duration at the weekend to ‘catch up’ on lost hours of sleep during the week (social jet lag), but this would be missed by the current measurement method for sleep duration. Was there a rationale for using only weekday sleep? If the choice was driven by the data available then this should be acknowledged as a limitation.

**Response: **Thank you for highlighting this. In children the measure is week day sleep and in mothers the measure is work day sleep. We acknowledge that both of these measures have limitations, for example, as you point out, chronotype could be an issue with weekday sleep. However, as this measure is used in children, this is likely to be less of an issue than in adults, and we hoped to capture routine sleep, hence why we have not used sleep on weekends (which was also available), which may vary more than weekday sleep. For the mothers, the limitation is slightly different, in that it only applies to those that work but it was the only measure available and again probably better than non-work days for capturing routine sleep. We have added the following text to the discussion (paragraphs 5 and 6):

“Small effect sizes may also reflect measurement error and quality of the parent-reported outcome measures. Self-reported sleep duration is subjective and therefore there will be some measurement error introduced when reporting average sleep duration, so the effect sizes we report here may underestimate the true correlations. This measurement error would also impact the parent-reported measures of sleep in their children, though we opted to use child’s sleep duration on weekdays only and not on weekends to capture routine sleep patterns.

Although we combined measures of sleep duration from several different ages, which is likely to have reduced error that was not shared across measurement occasions, sleep duration in the mothers is reported at a single time point and is unlikely to be consistent throughout the life course. The question for mother’s sleep duration also asks specifically about sleep on work days which may result in a bias if individuals who do not work did not answer this question. However, using data from another question asking, “Have you had any jobs or regular voluntary work in the past year?”, we found that of those who responded “yes” to this question, 98.64% responded to the question about sleep on work days. Of those who responded “no” to this question, 94.65% responded to the question about sleep on work days. These are quite similar response rates and although the latter is slightly smaller, we believe that this impact is negligible. More reliable measures of sleep duration are not currently available in the ALSPAC cohort, however, future studies comparing self-report measures to objective measures (such as accelerometer-derived sleep estimates 22) may help to estimate the potential biases in our results. Similarly, although we have reported results for sleep duration, we have no direct measure of sleep quality, so the marginally lower effect size for duration of sleep may index greater disruption in sleep architecture that could have important effects on psychopathology.”

Results:

- I think the phrasing for the result of ‘sleep duration in mothers’ may be unintentionally misleading. Whilst the effect size may be similar, the confidence intervals are wide, suggesting the effect size is imprecisely estimated (as the authors acknowledge). At the moment the manuscript reads as if increasing the sample size would improve the evidence in support of the association. It wouldn’t necessarily, but it would allow estimation of the effect with greater precision.

**Response: **Thank you for highlighting this. We have edited the sleep duration in mothers results paragraph to avoid misleading the reader:

“The estimated effect size of the association between schizophrenia polygenic risk score and sleep duration in mothers was similar to that seen in children, although confidence intervals are wide, so evidence in support of this association was weak (β=-49.97 seconds; 95% CI: −150.48, 50.55; p=0.33) ( Table 2). The smaller sample size in the mothers’ cohort has most likely led to lower power and therefore less precise estimates. An increased sample size would allow for estimation of this effect with greater precision.”

Discussion:

- There is little discussion about the result linking nightmares and schizophrenia. It would be helpful to discuss the size of the effect, and contextualise the result within relevant existing literature linking nightmares with psychosis.

**Response: **We have added the following text to the second paragraph of the discussion to add some more context to the nightmares and schizophrenia relationship:

“In addition, finding a small to moderate association between greater polygenic risk of schizophrenia and increased risk of nightmares supports previous phenotypic observations between nightmares and psychotic experiences suggesting the presence of nightmares may be an early risk indicator for psychosis ^4, 13, 25^ and suggesting that this relationship could be partly genetically mediated.”

 

- A study which may help address whether the results are due to pleiotropy is an experimental study by Reeve *et al.* (2018 ^1^) which manipulated sleep duration and assessed the impact on psychotic like experience. 

**Response: **We thank the reviewer for suggesting this study and we have added a reference to this, as indicated in the text below. This evidence of a causal association supports the idea that the genetic correlation may be due to a causal pathway rather than direct pleiotropy.

“For example, the findings could be due to horizontal pleiotropy, whereby genetic variants associated with schizophrenia are also independently associated with sleep phenotypes. The findings could also be due to causality, where either liability to schizophrenia causes disruption to sleep, or sleep disruption causes schizophrenia and so variants associated with sleep phenotypes are captured by the schizophrenia GWAS, which seems to be supported by experimental evidence ^23^. It is also possible that the polygenic risk score for schizophrenia may capture a shared risk factor for both schizophrenia and sleep disruption, e.g. urbanicity, and so the results represent the effect of this unknown shared risk factor on both phenotypes. Finally, associations may also be induced by population stratification where spurious results arise due to differences in genetic backgrounds between the discovery and target samples (although this is less likely due to restriction to those with a European ancestry). However, we also acknowledge that given that the effects detected in the current study are small, investigating underlying mechanisms between sleep factors and schizophrenia would be difficult. Objective sleep measures and more complete knowledge of the genetic variants related to sleep and schizophrenia would likely increase power to explore mechanisms.”

- Given evidence that there are differing levels of heritability for individual psychotic experiences, is the research limited by the fact that it has assessed risk for schizophrenia as a whole?

**Response: **This is an interesting point to consider and we have added the following text to indicate this limitation (paragraph 7 of discussion):

“As schizophrenia is highly heritable, better-powered GWAS will inevitably identify further variants and more accurately estimate genetic effect sizes, resulting in polygenic scores that account for a greater proportion of the variance. In addition, in our study we were unable to assess individual domains of schizophrenia, for example hallucinations, delusions or negative symptoms, as there are currently no available well powered GWAS for these outcomes. Future studies that reduce measurement error through objective measurements of sleep and more comprehensive polygenic risk scores that, for example, index specific symptom domains as well as disorder, will likely estimate more accurate and larger effect sizes than those we report here. As such, it is probably unwise to rule out clinical relevance on the basis of current effect size estimates.”

# Peer review: Reviewer response for version 1

**DOI:** 10.21956/wellcomeopenres.16430.r34686

**Published:** 2019-02-22

**Authors:** Victoria Garfield

**Affiliations:** 1 MRC Unit of Lifelong Health and Ageing at UCL, Institute of Cardiovascular Science, London, UK

This study used data from the well-known ALSPAC cohort to investigate whether there is shared genetic aetiology between sleep duration and nightmares, and schizophrenia liability, as sleep disturbances are common in schizophrenia. The authors used data from both mothers and children and implemented well-established methods for genetic analyses. Overall, this study is interesting, clearly written and well-conducted, but there are some important concerns that need to be addressed before indexing.

**Major points:**

1. I am not sure about the GWAS data that has been used for creating the schizophrenia polygenic risk scores, although I am aware that this is the best that is available. In particular, I am unsure of how valid it is here to weight child polygenic risk scores by effects from a (case-control) GWAS in adults, particularly for a disorder like schizophrenia, which is also much more likely to be diagnosed in adulthood. Could the authors comment on this and in their response, detail whether this was given any consideration and whether they think this is important? If they believe it not to be as important or have affected any of the results, I think an explanation should be provided.
2. There is no real acknowledgement in the Discussion section on the potential limitations of having used subjective sleep data for both mothers and children. Regarding the data in mothers, I know that all that was available was one timepoint, but it is important to acknowledge and briefly discuss that self-reported sleep data may be prone to error and some bias. There are plenty of studies to cite which show that the agreement between subjective and objective sleep duration data in adults is, at best, modest. Furthermore, the sleep data in mothers was only available when they were aged around 40 years, which is also important to acknowledge, as sleep duration changes throughout the life course and for example, mothers who have young children are less likely to have ‘normal’ sleep patterns compared to mothers who have older children or no children. Also, the question on sleep duration only assessed weekday sleep, which is another important thing to acknowledge and discuss. However, my understanding was that during this data sweep (~40 years) mothers were asked about both weekday and weekend sleep and that a weighted average was then taken. I also thought that there were sleep and genetic data available on up to ~7000 ALSPAC mothers at this age, so 2,302 seems much smaller. Could the authors please comment on this, as well as the above points on self-reported sleep?  

In relation to the child sleep data, it is also crucial to acknowledge that parent-reported data can be problematic (it was very quickly glossed over in the Discussion) and may also not agree with data collected via objective methods, such as actigraphy. This is because parents may misreport bedtimes and time they woke up, particularly if their child does not follow the most conventional bedtime, for example. Another important thing to consider here is that the question only asked about bedtime and time they awoke on *weekdays* and we know that as children get older their bedtimes change and they are more likely to go to bed at a different time during the weekend and potentially, wake up at different times.

 In general, it is important to acknowledge that if subjective and objective sleep measures correlate poorly then results could be different if objective measures were analysed.

**Introduction:**

1. Could the authors provide the age range or mean age of the twins in the previous TEDS study?

**Methods:**

1. Page 4: sleep duration section – it says that mothers were asked about how long they ‘slept for on **work** days’, should this be ‘week’ days?
2. Page 4: Covariates – what is the justification for the (quite narrow) choice of covariates? I think this needs to be included, as there could be other important covariates to consider.
3. Page 4: PRs construction – please could the authors include a reference or very brief reason for the clumping threshold of 0.25, as well as what your window for inclusion was (in kb)?

 

**Results:**

1. I cannot see a table with any sample characteristics anywhere – could the authors please include a table with at the very least, mean (SD) sleep durations for children at each age, mean (SD) sleep duration for mothers, %males or females (for children), mean (SD) age? One important reason for including the mean sleep durations for children and mothers is to see whether they were similar to the recommended averages for their ages, in line with guidelines for sleep for both children and adults.
2. I think it’s slightly unclear exactly how the main PRS results were obtained in children – in the supplementary file there are results presented by age, but the main results presented in Table 2 in the main text are unclear in terms of the sample used in these analyses. Presumably, there were some children who were present at 4 years of age but then lost to follow-up? I think that this could be clearer in terms of who was included in which analyses.
3. Is it correct to say that adjusting for the mother’s PRS and finding very little effect of this on the results in children shows that the ‘association is not confounded by the mother’s *psychopathology*’, given that the term psychopathology is broad and does not only encompass schizophrenia? The mother’s PRS was only a schizophrenia PRS, so would adjusting for this not simply ensure that the results in children were not affected by the mother’s genetic risk of schizophrenia or similar?

 

**Discussion:**

1. Page 6: the authors say that we still do not know the exact mechanisms behind the associations that they found between schizophrenia and sleep measures. However, wouldn’t these be extremely difficult to determine for such small effects?
2. Page 6: Could the authors give an example of what sort of ‘third variable’ they are referring to when they say that the PGC GWAS may have ascertainment bias?
3. It might be of interest in future to also perform a similar study with fathers/other adult males, as effects could be different.
4. Also, only sleep duration was examined and other sleep phenotypes may have different associations with a schizophrenia PRS. There are large-scale GWAS of other sleep phenotypes available too.
5. The authors have briefly acknowledged that the effect sizes they observed are small. They are, indeed, very small and a couple of the results have very wide 95% confidence intervals. In light of these very small effect sizes, could the authors comment, in the Discussion section, on the potential clinical meaning of these?

**Supplementary file:**

1. Could the authors please include the R ^2 ^values in each table for each PRS at every threshold? I think it would be informative for the reader to see how the R ^2 ^varied throughout the different models.

I confirm that I have read this submission and believe that I have an appropriate level of expertise to confirm that it is of an acceptable scientific standard, however I have significant reservations, as outlined above.

# Response

**Published:** 2019-08-13

**Authors:** Zoe Reed

**Affiliations:** University of Bristol, UK

**Major points:**

**1. **I am not sure about the GWAS data that has been used for creating the schizophrenia polygenic risk scores, although I am aware that this is the best that is available. In particular, I am unsure of how valid it is here to weight child polygenic risk scores by effects from a (case-control) GWAS in adults, particularly for a disorder like schizophrenia, which is also much more likely to be diagnosed in adulthood. Could the authors comment on this and in their response, detail whether this was given any consideration and whether they think this is important? If they believe it not to be as important or have affected any of the results, I think an explanation should be provided.

**Response: **Thank you for raising this point; we agree that we should justify this. Our primary aim here was specifically to look at whether genetic liability to develop schizophrenia in adulthood is related to sleep in childhood, so we used the best available genetic predictors of adult schizophrenia. But we strongly agree with the general point that the relationship between DNA variants and outcomes can be different at different ages (some of us have previously published on this e.g. Haworth and Davis, 2014), so if we were particularly interested in liability to childhood schizophrenia then weightings from an adult GWAS might not be appropriate. We have added clarification on why we constructed polygenic risk scores in children to the discussion (paragraph 1 of discussion):

“In this study we used summary statistics from a schizophrenia GWAS of an adult, clinical sample to construct polygenic risk scores in children and adults in the aim of understanding how schizophrenia genetic liability manifests in childhood. Our results demonstrate, for the first time with molecular genetic data, that greater polygenic risk for schizophrenia is associated with shorter sleep duration in both childhood and adulthood, and we report a new association between greater polygenic risk of schizophrenia and increased risk of nightmares in early life.”

Haworth CMA and Davis OSP (2014) From observational to dynamic genetics. Front. Genet. 5:6. doi: 10.3389/fgene.2014.00006

**2.** There is no real acknowledgement in the Discussion section on the potential limitations of having used subjective sleep data for both mothers and children. Regarding the data in mothers, I know that all that was available was one timepoint, but it is important to acknowledge and briefly discuss that self-reported sleep data may be prone to error and some bias. There are plenty of studies to cite which show that the agreement between subjective and objective sleep duration data in adults is, at best, modest. Furthermore, the sleep data in mothers was only available when they were aged around 40 years, which is also important to acknowledge, as sleep duration changes throughout the life course and for example, mothers who have young children are less likely to have ‘normal’ sleep patterns compared to mothers who have older children or no children. Also, the question on sleep duration only assessed weekday sleep, which is another important thing to acknowledge and discuss. However, my understanding was that during this data sweep (~40 years) mothers were asked about both weekday and weekend sleep and that a weighted average was then taken. I also thought that there were sleep and genetic data available on up to ~7000 ALSPAC mothers at this age, so 2,302 seems much smaller. Could the authors please comment on this, as well as the above points on self-reported sleep?  

In relation to the child sleep data, it is also crucial to acknowledge that parent-reported data can be problematic (it was very quickly glossed over in the Discussion) and may also not agree with data collected via objective methods, such as actigraphy. This is because parents may misreport bedtimes and time they woke up, particularly if their child does not follow the most conventional bedtime, for example. Another important thing to consider here is that the question only asked about bedtime and time they awoke on *weekdays* and we know that as children get older their bedtimes change and they are more likely to go to bed at a different time during the weekend and potentially, wake up at different times.

In general, it is important to acknowledge that if subjective and objective sleep measures correlate poorly then results could be different if objective measures were analysed.

**Response: **Thank you for highlighting these potential limitations. In response, we have added the following text to the discussion (paragraphs 5 and 6 of discussion):

“Small effect sizes may also reflect measurement error and quality of the parent-reported outcome measures. Self-reported sleep duration is subjective and therefore there will be some measurement error introduced when reporting average sleep duration, so the effect sizes we report here may underestimate the true correlations. This measurement error would also impact the parent-reported measures of sleep in their children, though we opted to use child’s sleep duration on weekdays only and not on weekends to capture routine sleep patterns.

Although we combined measures of sleep duration from several different ages, which is likely to have reduced error that was not shared across measurement occasions, sleep duration in the mothers is reported at a single time point and is unlikely to be consistent throughout the life course. The question for mother’s sleep duration also asks specifically about sleep on work days which may result in a bias if individuals who do not work did not answer this question. However, using data from another question asking, “Have you had any jobs or regular voluntary work in the past year?”, we found that of those who responded “yes” to this question, 98.64% responded to the question about sleep on work days. Of those who responded “no” to this question, 94.65% responded to the question about sleep on work days. These are quite similar response rates and although the latter is slightly smaller, we believe that this impact is negligible. More reliable measures of sleep duration are not currently available in the ALSPAC cohort, however, future studies comparing self-report measures to objective measures (such as accelerometer-derived sleep estimates ^22^) may help to estimate the potential biases in our results. Similarly, although we have reported results for sleep duration, we have no direct measure of sleep quality, so the marginally lower effect size for duration of sleep may index greater disruption in sleep architecture that could have important effects on psychopathology.”

In addition, we thank you for highlighting the sample size differences in the ALSPAC mothers analysis sample. This was due to an error in a script which resulted in more mothers being removed from the analyses than intended. Therefore, we have rerun all analyses with the correct sample size (n = 4,906) and have updated the results to reflect this. We do not observe any major differences in the results and conclusions for these analyses. However, we note that the sample size is still smaller than 7,000 individuals due to the number of individuals who have both phenotypic and genetic data.

**Introduction:**

**1.** Could the authors provide the age range or mean age of the twins in the previous TEDS study?

**Response: **The age range or mean age are not specifically provided in the TEDS paper, but data was collected when all children were around age 16 years. We have added this to the introduction in the relevant section (paragraph 2 of the introduction):

“A recent report from the Twins Early Development Study (TEDS) demonstrated that psychotic-like experiences and sleep disturbances in adolescence, around the age of 16 years, share genetic influences ^7^.”

**Methods:**

**1.** Page 4: sleep duration section – it says that mothers were asked about how long they ‘slept for on **work** days’, should this be ‘week’ days?

**Response: **This is correctly labelled as work days. I have added this potential limitation to the discussion (see above), as this may result in individuals that do not work not responding. However, we have checked this using another question, in the same questionnaire, asking “Have you had any jobs or regular voluntary work in the past year?”. We found that of those who responded “yes” to this question, 98.64% responded to the question about sleep on work days. Of those who respnded “no” to this question, 94.65% responded to the question about sleep on work days. These are quite similar response rates and although the latter is slightly smaller we believe that this impact is negligible. We have added these percentages in the following text to the discussion (paragraph 6):

“The question for mother’s sleep duration also asks specifically about sleep on work days which may result in a bias if individuals who do not work did not answer this question. However, using data from another question asking, “Have you had any jobs or regular voluntary work in the past year?”, we found that of those who responded “yes” to this question, 98.64% responded to the question about sleep on work days. Of those who responded “no” to this question, 94.65% responded to the question about sleep on work days. These are quite similar response rates and although the latter is slightly smaller, we believe that this impact is negligible.”

**2. **Page 4: Covariates – what is the justification for the (quite narrow) choice of covariates? I think this needs to be included, as there could be other important covariates to consider.

**Response: **We have included just age and sex as covariates as other covariates would fall, temporally, between the polygenic score and the sleep outcomes and may act as mediators so adjusting for these could lead to misleading results. We have added the following text to the covariates section in the methods to clarify this:

“We did not control for covariates beyond age and sex within the analyses as these would fall temporally between the independent variables (polygenic scores) and outcome (sleep) and could therefore act as mediators. Adjusting for these potential mediators could lead to misleading results.”

**3.** Page 4: PRs construction – please could the authors include a reference or very brief reason for the clumping threshold of 0.25, as well as what your window for inclusion was (in kb)?

**Response: **We have used an R ^2^ clumping threshold of 0.25 in line with previous literature (e.g. St Pourcain *et al.*, 2018), but we agree there is no general consensus. We used a 500kb window for inclusion, and we have clarified this in the polygenic risk score construction section of the methods.

Pourcain BS, Robinson EB, Anttila V, Sullivan BB, Maller J, Golding J, *et al.* ASD and schizophrenia show distinct developmental profiles in common genetic overlap with population-based social communication difficulties. Mol. Psychiatry. Nature Publishing Group; 2018;23:263–70.

**Results:**

**1.** I cannot see a table with any sample characteristics anywhere – could the authors please include a table with at the very least, mean (SD) sleep durations for children at each age, mean (SD) sleep duration for mothers, %males or females (for children), mean (SD) age? One important reason for including the mean sleep durations for children and mothers is to see whether they were similar to the recommended averages for their ages, in line with guidelines for sleep for both children and adults.

**Response:** We agree, and we have added these sample characteristics to table 1.

**2. **I think it’s slightly unclear exactly how the main PRS results were obtained in children – in the supplementary file there are results presented by age, but the main results presented in Table 2 in the main text are unclear in terms of the sample used in these analyses. Presumably, there were some children who were present at 4 years of age but then lost to follow-up? I think that this could be clearer in terms of who was included in which analyses.

**Response: **For the main results in children for sleep duration we combined all time points into one stacked dataset and used a linear mixed-effects model with age nested within family ID as a random effect. This resulted in 28,138 data points included in that model. This information is included in the text of the methods section. We have also added this information to the table 2 legend to clarify this.

**3.** Is it correct to say that adjusting for the mother’s PRS and finding very little effect of this on the results in children shows that the ‘association is not confounded by the mother’s *psychopathology*’, given that the term psychopathology is broad and does not only encompass schizophrenia? The mother’s PRS was only a schizophrenia PRS, so would adjusting for this not simply ensure that the results in children were not affected by the mother’s genetic risk of schizophrenia or similar?

**Response: **Yes, this is correct, thank you for highlighting that. We have changed this to genetic liability to schizophrenia.

**Discussion:**

**1. **Page 6: the authors say that we still do not know the exact mechanisms behind the associations that they found between schizophrenia and sleep measures. However, wouldn’t these be extremely difficult to determine for such small effects?

**Response: **We have added the following text to the discussion (paragraph 8) to reflect this:

“However, we also acknowledge that given that the effects detected in the current study are small, investigating underlying mechanisms between sleep factors and schizophrenia would be difficult. Objective sleep measures and more complete knowledge of the genetic variants related to sleep and schizophrenia would likely increase power to explore mechanisms.”

**2.** Page 6: Could the authors give an example of what sort of ‘third variable’ they are referring to when they say that the PGC GWAS may have ascertainment bias?

**Response: **We have rephrased this in the paper to clarfy what we mean by this. To clarify, we mean that the polygenic risk score manifests as a shared risk factor for both schizophrenia and disordered sleep. This risk factor would be unknown, however this could be something like urbanicity which could increase risk of developing schizophrenia and disordered sleep. We have changed the text to the following in the discussion (paragraph 8 of discussion):

“For example, the findings could be due to horizontal pleiotropy, whereby genetic variants associated with schizophrenia are also independently associated with sleep phenotypes. The findings could also be due to causality, where either liability to schizophrenia causes disruption to sleep, or sleep disruption causes schizophrenia and so variants associated with sleep phenotypes are captured by the schizophrenia GWAS, which seems to be supported by experimental evidence ^23^. It is also possible that the polygenic risk score for schizophrenia may capture a shared risk factor for both schizophrenia and sleep disruption, e.g. urbanicity, and so the results represent the effect of this unknown shared risk factor on both phenotypes. Finally, associations may also be induced by population stratification where spurious results arise due to differences in genetic backgrounds between the discovery and target samples (although this is less likely due to restriction to those with a European ancestry).”

**3.** It might be of interest in future to also perform a similar study with fathers/other adult males, as effects could be different.

**Response: **We have added the following text to the discussion (paragraph 9 of discussion):

“It would also be interesting to see whether the same relationship between polygenic risk for schizophrenia and sleep duration is observed in adult males, as were only able to examine this in adult females here.”

**4.** Also, only sleep duration was examined and other sleep phenotypes may have different associations with a schizophrenia PRS. There are large-scale GWAS of other sleep phenotypes available too.

**Response:** We agree that it would be interesting to look at a range of sleep phenotypes, however, the sleep measures investigated were the best measures available in ALSPAC. Whilst it would be interesting to incorporate sleep GWAS data into this study to look at whether genetic risk for sleep duration increases risk of schizophrenia, we do not have a measure of diagnosis of schizophrenia within ALSPAC.

**5. **The authors have briefly acknowledged that the effect sizes they observed are small. They are, indeed, very small and a couple of the results have very wide 95% confidence intervals. In light of these very small effect sizes, could the authors comment, in the Discussion section, on the potential clinical meaning of these?

**Response: **We have added the following text to the discussion (paragraph 3 of the discussion), where we discuss the small effect size:

“Although the effect sizes we report imply limited clinical relevance, the aforementioned limitations on our measurement of both sleep and genetic risk for schizophrenia provide a practical upper bound for the effect size we could have found. As schizophrenia is highly heritable, better-powered GWAS will inevitably identify further variants and more accurately estimate genetic effect sizes, resulting in polygenic scores that account for a greater proportion of the variance. In addition, in our study we were unable to assess individual domains of schizophrenia, for example hallucinations, delusions or negative symptoms, as there are currently no available well powered GWAS for these outcomes. Future studies that reduce measurement error through objective measurements of sleep and more comprehensive polygenic risk scores that, for example, index specific symptom domains as well as disorder, will likely estimate more accurate and larger effect sizes than those we report here. As such, it is probably unwise to rule out clinical relevance on the basis of current effect size estimates.”

**Supplementary file:**

**1.** Could the authors please include the R ^2 ^values in each table for each PRS at every threshold? I think it would be informative for the reader to see how the R ^2 ^varied throughout the different models.

**Response: **These have been added for all tables for the different thresholds for sleep duration in children and mothers and for nightmares.
